# Mesocortical Dopamine Phenotypes in Mice Lacking the Sonic Hedgehog Receptor Cdon

**DOI:** 10.1523/ENEURO.0009-16.2016

**Published:** 2016-07-13

**Authors:** Michael Verwey, Alanna Grant, Nicholas Meti, Lauren Adye-White, Angelica Torres-Berrío, Veronique Rioux, Martin Lévesque, Frederic Charron, Cecilia Flores

**Affiliations:** 1 Department of Psychiatry, Douglas Mental Health University Institute, McGill University, Montreal, QC, Canada; 2 Molecular Biology of Neural Development, Institut de Recherches Cliniques de Montréal, Montreal, QC, Canada; 3 Department of Medicine, University of Montreal, Montreal, QC, Canada; 4 Department of Anatomy and Cell Biology, Department of Biology, Division of Experimental Medicine, McGill University, Montreal, QC, Canada; 5 Program in Neuroengineering, McGill University, Montreal, QC, Canada; 6 Department of Psychiatry and Neurosciences, Faculty of Medicine, Université Laval, Québec, QC, Canada; 7 Centre de Recherche Université Laval-Robert-Giffard, Université Laval, Québec, QC, Canada

**Keywords:** Cdon, dopamine, medial prefrontal cortex, sonic hedgehog, ventral tegmental area

## Abstract

Motivated behaviors and many psychopathologies typically involve changes in dopamine release from the projections of the ventral tegmental area (VTA) and/or the substantia nigra pars compacta (SNc). The morphogen Sonic Hedgehog (Shh) specifies fates of midbrain dopamine neurons, but VTA-specific effects of Shh signaling are also being uncovered. In this study, we assessed the role of the Shh receptor Cdon in the development of VTA and SNc dopamine neurons. We find that Cdon is expressed in the proliferating progenitor zone of the embryonic ventral midbrain and that the number of proliferating cells in this region is increased in mouse *Cdon^−/−^* embryos. Consistent with a role of Shh in the regulation of neuronal proliferation in this region, we find that the number of tyrosine hydroxylase (TH)-positive neurons is increased in the VTA of *Cdon^−/−^* mice at birth and that this effect endures into adulthood. In contrast, the number of TH-positive neurons in the SNc is not altered in *Cdon^−/−^* mice at either age. Moreover, adult *Cdon^−/−^* mice have a greater number of medial prefrontal cortex (mPFC) dopamine presynaptic sites, and increased baseline concentrations of dopamine and dopamine metabolites selectively in this region. Finally, consistent with increased dopamine function in the mPFC, we find that adult *Cdon^−/−^* mice fail to exhibit behavioral plasticity upon repeated amphetamine treatment. Based on these data, we suggest that Cdon plays an important role encoding the diversity of dopamine neurons in the midbrain, influencing both the development of the mesocortical dopamine pathway and behavioral outputs that involve this neural circuitry.

## Significance Statement

Sonic hedgehog signaling is involved in the specification and development of dopamine neurons in the ventral midbrain. Here we demonstrate that the Shh receptor Cdon plays a role in the development of dopamine neurons in the ventral tegmental area. Moreover, this effect of Cdon is selective to the dopamine neurons that project to the medial prefrontal cortex. Adult mice that lack Cdon also fail to show amphetamine-induced behavioral plasticity. Our findings show that the Cdon receptor is important in encoding the diversity of dopamine neurons in the midbrain, influencing both the development of the mesocortical dopamine pathway as well as behavioral outputs that involve this neural circuitry.

## Introduction

Midbrain dopamine neurons are involved in diverse behavioral and psychological processes, and alterations in their development can have implications that range from motor deficits to psychopathology (Björklund and Dunnett, 2007; Blesa and Przedborski, 2014; Volkow and Morales, 2015). Dopamine neurons in the ventral tegmental area (VTA) and substantia nigra pars compacta (SNc) share basic neurochemical similarities, but increasing evidence shows that they are heterogeneous and that their physiological properties vary in a target-dependent manner (Roeper, 2013). Likewise, developmental mechanisms that define the segregation of VTA and SNc dopamine neurons, and the unique cortical and striatal projections that they make, have also begun to emerge (Van den Heuvel and Pasterkamp, 2008; Anderegg et al., 2015; Bissonette and Roesch, 2016).

One example is the sonic hedgehog (Shh) signaling pathway, which is involved in the specification of dopamine cell fate (Hynes et al., 1995; Wang et al., 1995; Wallén and Perlmann, 2003) and acts as a chemoattractant that promotes the rostral projections of these neurons (Hammond et al., 2009). In order to activate the Shh pathway, Shh binds to Patched1 (Ptch1), which leads to Smoothened disinhibition and the activation of Gli transcription factors. Shh signaling acts in two phases during the specification of dopaminergic neurons; during the first phase, notochord-derived Shh initiates the specification of the ventral midbrain, including the progenitors of dopamine neurons. During the second phase, Shh is expressed by dopamine neuron progenitors themselves, and the duration of Shh expression contributes to their fate decisions and their segregation between the VTA and SNc (Blaess et al., 2011; Hayes et al., 2011, 2013). Therefore, the fate decisions of dopamine progenitors and the numbers of dopamine cells in the VTA and/or SNc are differentially influenced by Shh signaling, depending on how and at what developmental time the Shh signaling pathways is manipulated (Blaess et al., 2011; Hayes et al., 2011, 2013; Kabanova et al., 2015). As a result, variations in Shh signaling, at selective developmental times, must influence behaviors in adulthood that depend on mesocorticolimbic and/or nigrostriatal dopamine pathways. Such variations in Shh signaling might therefore be involved in distinct psychopathologies.

Cell adhesion molecule-related/downregulated by oncogenes (Cdon) is a Ptch1 coreceptor that binds Shh (Okada et al., 2006) and modulates pathway activity (Okada et al., 2006; Allen et al., 2011; Yam and Charron, 2013). The role of Cdon in segregating dopamine neurons between the VTA and SNc, and its potential impacts on behavior, have never been explored. Here we show that Cdon is expressed in the embryonic ventral midbrain dopaminergic progenitors. Based on this finding, we hypothesized that Cdon could mediate some of the general, and possibly region-specific (i.e., VTA vs SNc) effects of Shh on the development of the dopamine system and, in turn, influence dopamine-mediated behaviors in adulthood. To this end, we compared wild-type (WT) and *Cdon^−/−^* embryos at embryonic day 12.5 (E12.5), and identified a potential role for Cdon in the regulation of proliferation in the midbrain dopaminergic progenitors. Consistent with a putative increase in the proliferation of dopamine progenitors in *Cdon^−/−^* mice, we observed an increase in the number of dopamine neurons in *Cdon^−/−^* mice immediately after birth and in adult life. Importantly, this increase was specifically observed in the VTA. Next, we examined dopamine concentrations in forebrain regions that receive dopamine projections from the VTA or the SNc and found increased levels of dopamine and dopamine metabolites in the medial prefrontal cortex (mPFC), but not in the nucleus accumbens (NAcc) and dorsal striatum (DS), of adult *Cdon^−/−^* mice. Furthermore, we found that adult *Cdon^−/−^* mice have an increased number of mPFC dopamine presynaptic sites. To determine potential behavioral consequences of these neuroanatomical and neurochemical changes, we evaluated amphetamine-induced behavioral plasticity in adult *Cdon^−/−^* mice and found important deficits. These findings show that Cdon is important in the development of VTA dopamine neurons, particularly those projecting to the mPFC, and in turn influences adult behaviors that are dependent on these pathways.

## Materials and Methods

### Animal housing and breeding

All animal housing, experiments, and procedures were approved by the Animal Care Committee at the Douglas Mental Health University Institute, McGill University (Montreal, QC, Canada) and at the Institut de Recherches Cliniques de Montréal, and were all performed in accordance with the guidelines set out by the Canadian Council of Animal Care (http://www.ccac.ca). *Cdon^−/−^* mice (Okada et al., 2006) were generated by a gene trap vector that targeted the transmembrane domain of Cdon (Friedel et al., 2005) and were backcrossed with C57BL/6 mice for at least 10 generations. Experimental *Cdon^−/−^* mice were generated by crossing *Cdon^+/−^* breeders. Male and female offspring were pooled for embryonic and postnatal day 0 (P0) studies, as well as in the quantification of tyrosine hydroxylase (TH)-positive varicosities in the mPFC. All other experiments used only male mice.

### Immunohistochemistry and stereological analyses

#### Tissue preparation and sectioning

Embryos and P0 pups were dissected, postfixed in a 4% paraformaldehyde solution (24 h, 4°C), cryoprotected in a sucrose solution (24 h, 15% sucrose, 4°C), then snap frozen in optimal cutting temperature medium (Tissue-Tek, Cedarlane) and stored at −80°C until slicing. Embryos (14 μm sections) and P0 (35 μm sections) pups were sliced on a cryostat (CM3050S, Leica), sections were collected on charged Superfrost Slides (Fisherbrand), and stored at −80°C until use. Adult male mice (postnatal day 75 ±15) were deeply anesthetized with sodium pentobarbital (>75 mg/kg, i.p.), perfused transcardially with ∼50 ml of 0.9% saline followed by ∼50 ml of 4% paraformaldehyde. Brains were dissected, postfixed overnight (4°C), and sliced on a Vibratome (35 μm sections; Leica). Serial coronal sections were stored free-floating in Watson’s cryoprotectant at −20°C until processing (Watson et al., 1986).

#### Immunohistochemistry

Immunohistochemistry and immunofluorescent staining was performed (Okada et al., 2006; Manitt et al., 2010, 2011; Mille et al., 2014) with anti-TH mouse (1:1000; MAB318, Millipore Bioscience Research Reagents), anti-TH rabbit (1:1000; MAB152, Millipore Bioscience Research Reagents), anti-Ki67 mouse (1:250; catalog #550609, BD Biosciences), anti-Cdon goat (1:500; AF2429, R&D Systems), and anti-β-galactosidase (β-Gal) rabbit (1:1000; catalog #0855976, MP Biologicals) antibodies. Antigen retrieval was used prior to all embryonic labeling, and Alexa Fluor 488-, Alexa Fluor 555-, or Alexa Fluor 643-conjugated secondary antibodies (Molecular Probes) were used for immunofluorescence. For P0 and adult stereology experiments that quantified TH-positive cells in the VTA and SN, a 3% hydrogen peroxide pretreatment was used to inactivate endogenous peroxidases, and a 3,3'-diaminobenzidine kit was used according to manufacturer instructions (PK-4000 ABC kit, SK-4100 DAB kit, Vector Laboratories). For stereological quantification of TH-positive varicosities in the mPFC, TH was visualized with an Alexa Fluor 555-conjugated secondary antibody.

#### Microscopy and analysis

Serial coronal sections of embryos and adult brains were examined with Leica DM4000 and DM6000 microscopes with an Orca ER CCD camera (Hamamatsu) using Volocity (PerkinElmer) or Stereo Investigator (MBF Bioscience) software. In order to avoid including mice with signs of holoprosencephaly (HPE), we inspected for HPE on the live/intact mouse or embryo and performed a careful and systematic morphological analysis under the microscope. Specifically, all embryos and mice were inspected for any signs of cebocephaly and incomplete forebrain clefting (Zhang et al., 2006). At P0, we observed a single instance of malformed olfactory bulbs, which is another sign of HPE and led to the exclusion of this mouse (Zhang et al., 2006). Finally, across all ages, we examined carefully for enlarged or malformed ventricles. We also verified that mice did not show tooth malformations, which is another symptom of Cdon-associated HPE (Cole and Krauss, 2003), and weighed mice regularly to identify possible difficulties eating. All the adult *Cdon^−/−^* mice included in the study had weights that were similar to those of the WT littermates.

Embryonic TH and Ki67 immunoreactivity was counted manually with ImageJ software, was averaged for at least two sections/level/embryo, and was analyzed by two-way ANOVA_Genotype×Level_. Stereology was performed to quantify the number of TH-positive cell bodies in the VTA and SNc, and the number of TH-positive varicosities in the mPFC (Manitt et al., 2013; Daubaras et al., 2014). Briefly, the number of TH-positive cells was counted in the VTA and SNc of *Cdon^−/−^* mice and WT littermate controls at P0 and P75 ± 15 with a stereological fractionator sampling design (West et al., 1991), and Stereoinvestigator software (MBF Bioscience). The VTA- and SNc-containing sections ranged from Plate 54 to Plate 57 of the mouse brain atlas (Franklin and Paxinos, 2007). The counting frame (75 × 75 μm) and grid size (150 × 150 μm) were chosen manually. Counting was performed using every other brain section. A guard zone of 5 μm at the top and bottom of the section was used, and the coefficient of error was <0.1 in all animals studied, and the experimenter was blind to experimental groups.

To obtain a measure of the presynaptic density of dopamine synapses in the pregenual mPFC, TH-positive varicosities were quantified in this structure. TH-positive varicosities are sites of putative synapses with a dendritic spine or shaft (Séguéla et al., 1988), and are where neurotransmitter synthesis, release, and reuptake generally occur (Benes et al., 1996). Consistent with previous neuroanatomical studies (Manitt et al., 2011; Reynolds et al., 2015), and because of the lateralization of the dopamine system, we only obtained counts from the right hemisphere. Using Stereo Investigator software (MBF Bioscience), we made stereological quantifications of the volume and the number of TH-positive varicosities in the cingulate (Cg), prelimbic (PL), and infralimbic (IL) subregions of the mPFC. These subregions were delineated according to plates 14–18 of the mouse brain atlas (Paxinos and Franklin, 2008), and the contours of the dense TH-positive innervation within each subregion were traced at 5× magnification using a Leica DM4000 microscope. An unbiased counting frame (25 × 25 µm) was superimposed on each contour, and counts were made at regular predetermined intervals (175 × 175 µm). All counting of varicosities was performed at 100× magnification on 6 of the 12 sections contained within the rostrocaudal borders of our region of interest (1:2 series). Guard zones (4 µm) and an optical dissector (10 µm) were used. We used the Cavalieri method in Stereo Investigator (MBF Bioscience) to assess the volume of TH-positive fiber innervation (µm^3^), and the optical fractionator probe was then used to count TH-positive varicosities. The Gundersen coefficient of error was <0.15 for all regions of interest in all sampled brains.

### Analysis of dopamine and dopamine metabolite concentrations in rostral targets of midbrain dopamine neurons

#### Tissue preparation

As described previously (Grant et al., 2009, 2014), mice were decapitated, and their brains were rapidly dissected and snap frozen in 2-methylbutane (Fisher Scientific) on dry ice. Brains were then sliced on a cryostat and 0.5 mm punches (catalog #18035-50, Fine Science Tools) were taken bilaterally to dissect the pregenual mPFC (pooling Cg, PL, and IL subregions) and NAcc (including both shell and core), and a 1.0 mm punch was taken DS (dorsolateral portion); then all samples were frozen at −80°C until use.

#### High-performance liquid chromatography

Levels of dopamine, 3,4-dihydroxyphenylacetic acid (DOPAC) and homovanillic acid (HVA) in the DS, NAcc, and mPFC were assessed using high-performance liquid chromatography (HPLC; Grant et al., 2007). Briefly, brain punches from each area were homogenized in a 0.1 m phosphate buffer, centrifuged, the supernatant was then removed and filtered for HPLC testing, and the pellet was resuspended for quantification of the protein content (Bicinchoninic Acid Kit, catalog #P123225, Thermo Scientific). The HPLC assay for dopamine, DOPAC, and HVA was performed with an EZChrom Chromatography System (Scientific Software Inc). Dopamine and metabolites were detected and quantified with a Coulochem III detector, and concentrations were calculated from peak height comparisons with known amounts of injected pure standards (Sigma-Aldrich). Significance levels used to evaluate statistical differences were adjusted using the Holm-Bonferroni’s sequentially rejective procedure (Holm, 1979).

### Behavioral testing

#### Locomotor activity testing

As described previously (Grant et al., 2009; Yetnikoff et al., 2010), locomotor activity was measured by an infrared system that monitors total horizontal distance travelled within a defined period of time (AccuScan Instruments). On day 1, mice were habituated to the locomotor chambers for 15 min. On day 2, following a 15 min habituation period, mice were habituated to the injection procedure with an intraperitoneal injection of saline, and locomotor activity was recorded for 30 min. On day 3, after habituation, mice were given 2.5mg/kg (i.p.) d-amphetamine, and locomotor activity was monitored for another 90 min. Next, all mice were given 4 mg/kg d-amphetamine every other day, for a total of five additional injections, delivered on days 5, 7, 9, 11, and 13. Finally, on day 21, after 8 d of drug abstinence in their home cages, mice were tested again with 2.5 mg/kg d-amphetamine (see Fig. 7D, diagram illustrating this schedule). Differences between the locomotor activity induced by the first dose of amphetamine (day 3) and the last dose of amphetamine (day 21) represent a form of behavioral plasticity known as locomotor sensitization (Stewart and Badiani, 1993; Pierce and Kalivas, 1997).

#### Prepulse inhibition

As described previously (Grant et al., 2007), prepulse inhibition (PPI) was assessed using sound-attenuated startle chambers (SR-LAB, San Diego Instruments) containing a clear restraining tube that housed the animal throughout the testing session and background white noise (70 dB) was delivered continuously. Prior to each session, all chambers were calibrated to ensure consistent sensitivity and stable sound levels between testing boxes. A 120 dB pulse induced a startle response in mice, which was recorded by computer, and an average of 65 readings was taken at 1 ms intervals after the startle pulse. Each prepulse was delivered 100 ms before the acoustic startle, and lasted 20 ms. Within each session, there was a total of 54 trials in a pseudorandom order, which included 12 startle trials with no prepulse, 6 trials with prepulses at each volume (3, 5, 7, 10, 15, and 20 dB, above the 70 dB background noise), and 6 null trials where no acoustic startle was presented. The degree of PPI was then calculated as a percentage for each prepulse intensity: PPI% = 1 − (mean prepulse − mean null)/(mean startle − mean null)*100.

### Statistical analyses

All Student’s *t* tests, analyses of variance, and Bonferroni’s *post hoc* tests were performed using Prism 5 (GraphPad Software). For each figure and statistical test, *F* and *t* values are reported in Table 1. Specifically, in Figure 2, C and E, Student’s *t* test was used, and in Figure 2, D and F, two-way ANOVA_Genotype×Level_ was used. In Figure 3, stereological means were compared within each brain area by Student’s *t* test. In Figure 4, planned comparisons were made using the Holm–Bonferroni’s sequentially rejective procedure (Holm, 1979). In Figure 5, a two-way ANOVA_Genotype×Region_ was used. In Figure 6A–C, two-way ANOVA_Genotype×Time_ was used to compare groups over the duration of the test, and in Figure 6, E and F, two-way ANOVA_Genotype×Time_ was used with Bonferroni’s *post hoc* comparisons. In Figure 7, a two-way ANOVA_Genotype×ppvolume_ was used. All graphs illustrate the mean ± standard error.

**Table 1:** Statistical tests and values

Graph		Type of test	Statistical values
a. b. c. d. e. f. g. h. i. j. k. l. m. n. o. p.	Figure 2C Figure 2D Figure 2E Figure 2F Figure 3A (VTA) Figure 3A (SN) Figure 3B (VTA) Figure 3B (SN) Figure 4B (mPFC) Figure 4B (NAcc) Figure 4C (DS) Figure 5B Figure 5C Figure 5D Figure 6A Figure 6B Figure 6C Figure 6E Figure 6F Figure 7	Unpaired *t* test (two-tailed) ANOVA (genotype × level) ANOVA (genotype) ANOVA (level) Unpaired *t* test (two-tailed) ANOVA (genotype × level) ANOVA (genotype) ANOVA (level) Unpaired *t* test (two-tailed) Unpaired *t* test (two-tailed) Unpaired *t* test (two-tailed) Unpaired *t* test (two-tailed) DA unpaired *t* test (two-tailed) DOPAC unpaired *t* test (two-tailed) HVA unpaired *t* test (two-tailed) DA unpaired *t* test (two-tailed) DOPAC unpaired *t* test (two-tailed) HVA unpaired *t* test (two-tailed) DA unpaired *t* test (two-tailed) DOPAC unpaired *t* test (two-tailed) HVA unpaired *t* test (two-tailed) ANOVA (genotype × subregion) ANOVA (genotype) ANOVA (subregion) ANOVA (genotype × subregion) ANOVA (genotype) ANOVA (subregion) ANOVA (genotype × subregion) ANOVA (genotype) ANOVA (subregion) ANOVA (genotype × time) ANOVA (genotype) ANOVA (time) ANOVA (genotype × time) ANOVA (genotype) ANOVA (time) ANOVA (genotype × time) ANOVA (genotype) ANOVA (time) ANOVA (genotype × test) ANOVA (genotype) ANOVA (test) ANOVA (genotype × test) ANOVA (genotype) ANOVA (test) ANOVA (genotype × pp volume) ANOVA (genotype) ANOVA (pp volume)	*t*_(12)_ = 3.252, *p* = 0.0069^*^ F_(2,34)_ = 0.3468, *p* = 0.7094 *F*_(1,34)_ = 15.96, *p* = 0.0003^*^ F_(2,34)_ = 0.5282, *p* = 0.5944 *t*_(12)_ = 0.6990, *p* = 0.4979 *F*_(2,34)_ = 0.04603, *p* = 0.9551 *F*_(1,34)_ = 0.6569, *p* = 0.4233 *F*_(2,34)_ = 1.357, *p* = 0.2711 *t*_(6)_ = 3.655, *p* = 0.0105^*^ t_(6)_ = 1.399, *p* = 0.2114 *t*_(8)_ = 3.747, *p* = 0.0056^*^ t_(8)_ = 1.004, *p* = 0.3448 *t*_(15)_ = 5.482, *p* < 0.0001^*^ t_(15)_ = 6.529, *p* < 0.0001^*^ t_(15)_ = 0.02491, *p* = 0.9805 *t*_(15)_ = 0.8655, *p* = 0.4004 *t*_(15)_ = 0.3288, *p* = 0.7469 *t*_(15)_ = 0.6184, *p* = 0.5456 *t*_(15)_ = 0.1534, *p* = 0.8801 *t*_(15)_ = 0.4683, *p* = 0.6463 *t*_(15)_ = 1.245, *p* = 0.2321 *F*_(2,12)_ = 0.8166, *p* = 0.465 *F*_(1,12)_ = 10.13, *p* = 0.0079^*^ F_(2,12)_ = 21.25, *p* = 0.0001^*^ F_(2,12)_ = 0.5533, *p* = 0.5891 *F*_(1,12)_ = 1.431, *p* = 0.2547 *F*_(2,12)_ = 205.6, *p* < 0.0001^*^ F_(2,12)_ = 0.1561, *p* = 0.8572 *F*_(1,12)_ = 10.39, *p* = 0.0073^*^ F_(2,12)_ = 0.07934, *p* = 0.9242 *F*_(2,30)_ = 0.6911, *p* = 0.5088 *F*_(1,30)_ = 0.06015, *p* = 0.8096 *F*_(2,30)_ = 4.016, *p* = 0.0285^*^ F_(2,120)_ = 0.2720, *p* = 0.9739 *F*_(1,120)_ = 0.0003935, *p* = 0.9844 *F*_(8,120)_ = 83615, *p* < 0.0001^*^ F_(20,300)_ = 0.2543, *p* = 0.9996 *F*_(1,300)_ = 0.001303, *p* = 0.9717 *F*_(20,300)_ = 14.34, *p* < 0.0001^*^ F_1,15_= 4.882, *p* = 0.0431^*^ F_(1,15)_ = 2.417, *p* = 0.1409 *F*_(1,15)_ = 17.18, *p* = 0.0009^*^ F_(1,14)_ = 0.9707, *p* = 0.3412 *F*_(1,14)_ = 0.02339, *p* = 0.8806 *F*_(1,14)_ = 6.592, *p* = 0.0223^*^ F_(5,295)_ = 0.9344, *p* = 0.4589 *F*_(1,295)_ = 13.12, *p* = 0.0006^*^ F_(5,295)_ = 23.5, *p* < 0.0001^*^

## Results

### *Cdon* is expressed in proliferating midbrain dopamine progenitor cells at E12.5

**Figure 1.**
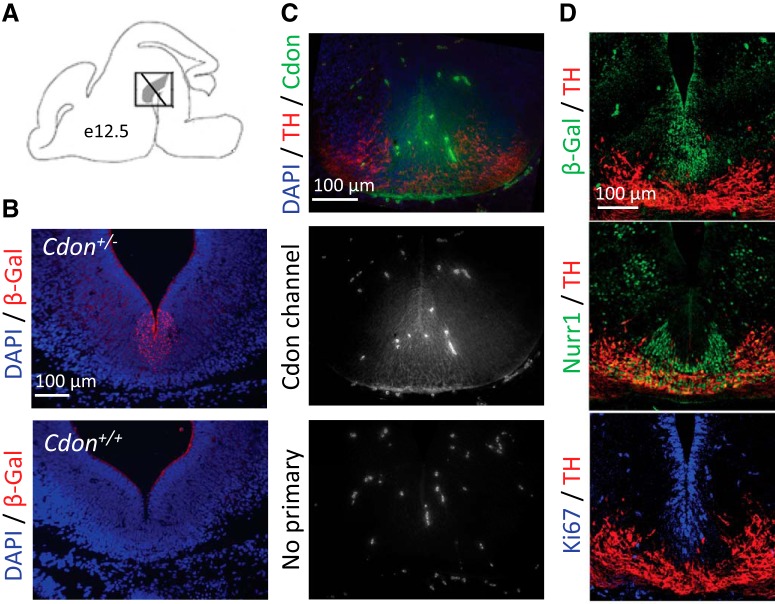
*Cdon* is expressed in proliferating progenitor cells of the ventral midbrain at E12.5. ***A***, Schematic illustration of a brain from E12.5 embryo showing the anteroposterior level used in the coronal sections shown in ***B–D***. ***B***, *Cdon^+/−^* embryos exhibit staining for β-Gal (Cdon) expression in the ventral midbrain (middle), which is not seen in WT negative control (bottom panel). ***C***, Cdon immunolabeling appears throughout the dopamine progenitor zone in the ventral midbrain of a WT embryo (top and middle), while a control section stained without primary antibody (bottom) has no such labeling. ***D***, β-Gal (Cdon) expression relative to TH (a marker of mature dopamine neurons), Nurr1 (a marker of immature postmitotic dopamine neurons), and Ki67 (a marker of proliferation) indicate that Cdon overlaps mainly with the proliferative Ki67-positive zone.

We assessed whether Cdon is expressed in the embryonic ventral midbrain. This was done using two complementary approaches in the ventral midbrain of E12.5 embryos (Fig. 1A). First, we used mice in which a gene encoding β-Gal was inserted in the *Cdon* gene by homologous recombination (Okada et al., 2006) and assessed β-Gal expression by immunofluorescence in *Cdon^+/−^* embryos. Periventricular and ventral β-Gal labeling was observed in a zone where progenitors proliferate and differentiate into dopamine neurons, as shown in Figure 1B (top). As a negative control, no β-Gal labeling was observed in *Cdon^+/+^* embryos under the same conditions (Fig. 1B, bottom). As a second approach, immunolabeling against the Cdon protein showed a very similar Cdon localization in the ventral midbrain of WT embryos (Fig. 1C, top and middle), confirming the results obtained using the β-Gal reporter. We next analyzed Cdon expression (using the β-Gal reporter) in the context of proliferating (Ki67), immature [nuclear receptor related 1 (Nurr1)], and mature dopamine neurons expressing TH in the ventral midbrain. We found that at E12.5, Cdon (β-Gal) expression was dorsal to, and did not overlap with, the TH-positive zone (Fig. 1D, top). Based on labeling in adjacent sections, there was a small overlap with immature dopamine neurons expressing Nurr1 but not TH (Fig. 1D, middle). However, Cdon (β-Gal) expression was strongest in the proliferative, Ki67-positive, progenitor zone (Fig. 1D, bottom). Therefore, at E12.5, Cdon is mostly expressed in the proliferating midbrain dopamine progenitors.

### Increased number of proliferating cells in the ventral midbrain of *Cdon^−/−^* embryos at E12.5

**Figure 2.**
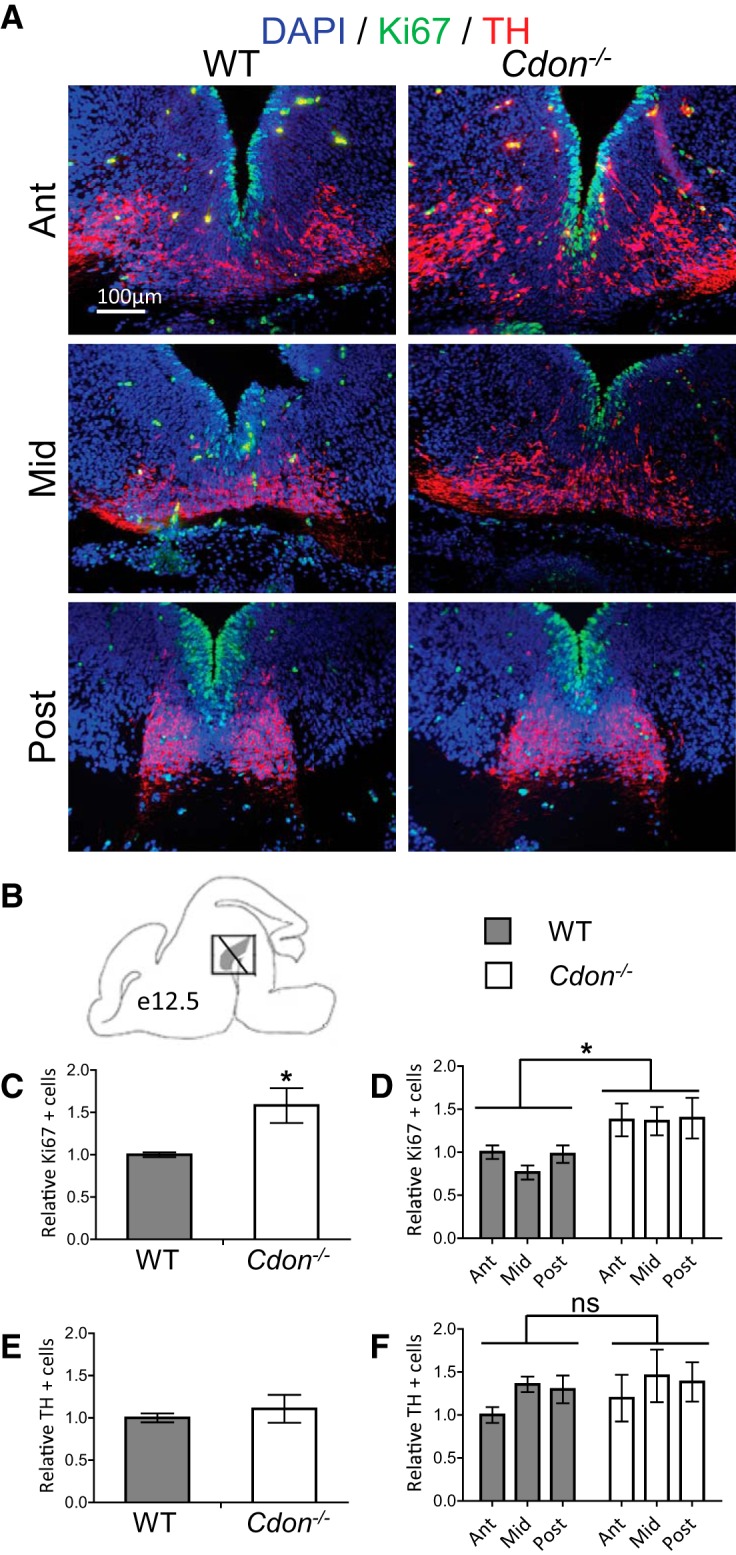
The number of proliferating cells in the ventral midbrain of *Cdon^−/−^* embryos is increased at E12.5. ***A***, Representative merged images of immunofluorescence for Ki67 (green), TH (red), and DAPI (blue) in coronal slices of the ventral midbrain of embryos at E12.5. ***B***, Schematic illustrating the coronal plane of analysis. ***C***, The total number of Ki67 immunoreactive cells was significantly increased in *Cdon^−/−^* embryos relative to WT controls (Student’s *t* test, *p* = 0.0069; Table 1, a), and, ***D***, this effect was seen across the anterior to mid-posterior extent of the ventral midbrain (ANOVA_Genotype×Level_, main effect of genotype, *p* = 0.0003; Table 1, b). ***E***, The total number of TH immunoreactive cells was similar between *Cdon^−/−^* embryos relative to WT controls (Student’s *t* test, *p* = 0.498; Table 1, c), and no genotype- or level-based effect was observed at, ***F***, anterior, mid, or posterior levels of the ventral midbrain (ANOVA_Genotype×Level_; Table 1, d). *n* = 6-8 embryos/group.

In order to assess the role of Cdon in the development of dopamine neurons, *Cdon^−/−^* and WT littermates were stained for Ki67 and TH at E12.5. Representative images of immunofluorescence are shown in Figure 2A, which are coronal sections from the ventral midbrain (Fig. 2B). *Cdon^−/−^* embryos exhibited a significant increase in the number of Ki67-positive cells on the ventricular border compared with WT littermates (Fig. 2C; unpaired *t* test, *p* = 0.0069; Table 1, a). Moreover, this effect was observed across the anterior–posterior axis (Fig. 2D; ANOVA_Genotype_, *p* = 0.0003; Table 1, b). This increase in Ki67 indicates that there is an increased level of proliferation of neural progenitors in the ventral midbrain of *Cdon^−/−^* embryos. In contrast, at the same embryonic stage, the number of TH-positive neurons was similar between genotypes (Fig. 2E; unpaired *t* test; Table 1, c). This was also true when individual levels of the anterior–posterior axis were investigated (Fig. 2F, ANOVA_Genotype×Level_; Table 1, d). These results indicate that inactivation of *Cdon* causes an increase in the number of proliferating progenitors, but that E12.5 could be still be too early to observe a change in the number of cells expressing TH.

### Postnatal increase in the number of TH-positive neurons in the VTA of *Cdon^−/−^* mice

**Figure 3.**
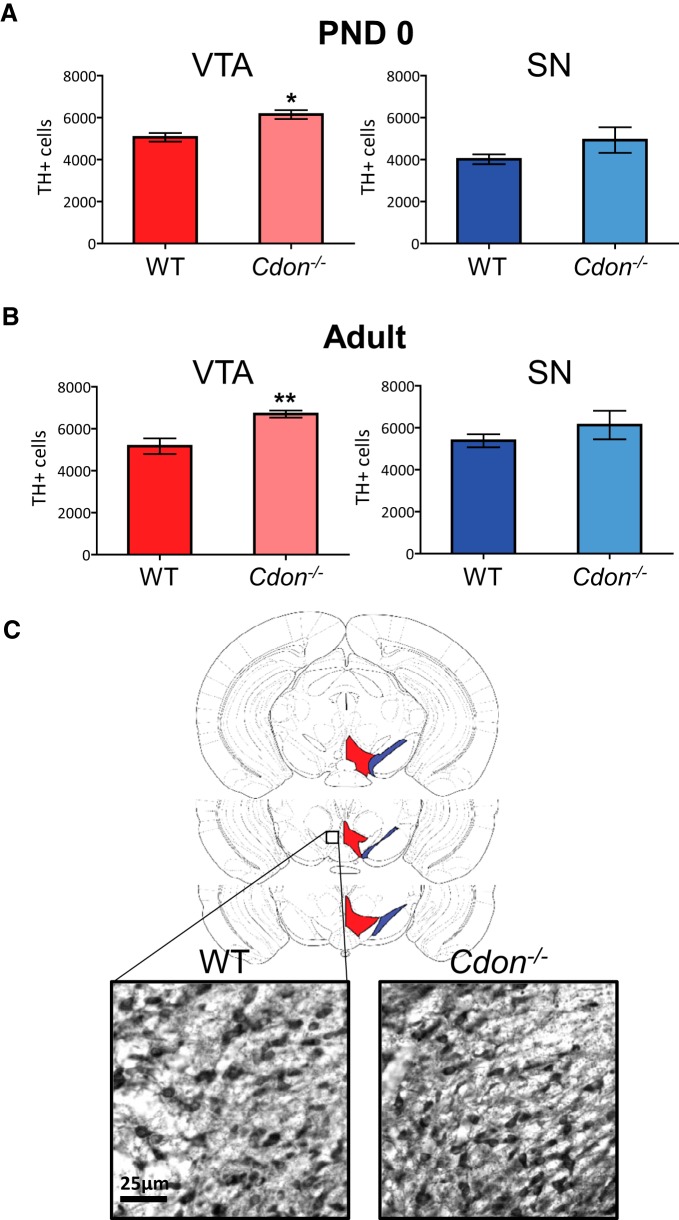
A greater number of TH-positive neurons in the VTA of *Cdon^−/−^* mice at birth and in adulthood. ***A***, ***B***, Total number of TH-positive neurons in the VTA (left, in red) and SN (right, in blue) in P0 (***A***) and adult mice (***B***) as measured by stereology. A greater number of TH-positive neurons were observed in the VTA of *Cdon^−/−^* mice compared to WT controls at birth (Student’s *t* test, *p* < 0.05; Table 1, e) and in adulthood (Student’s *t* test, *p* < 0.01; Table 1, f). ***C***, Mouse brain atlas illustrations showing the VTA and SN sections that were included in this analysis, and representative TH immunoreactivity in coronal sections of adult mice. *n* = 4-5 mice/group. *p < 0.05, **p < 0.01.

We next assessed whether this increase in progenitor proliferation leads to an increase in the numbers of dopamine neurons later in brain development and in adulthood. Stereological counts of TH-positive neurons in the VTA and SNc at P0 revealed a significant increase in the number of TH-positive cells in the VTA of *Cdon^−/−^* mice compared with WT littermates (Fig. 3A, left graph; Student’s *t* test, *p* = 0.01; Table 1, e). In contrast, the number of TH-positive cells in the SNc is not significantly changed between genotypes (Fig. 3A, right graph; Student’s *t* test; Table 1, e). Interestingly, the same pattern is observed in adult mice, where there are more TH-positive neurons in the VTA of adult *Cdon^−/−^* mice compared with WT littermates (Fig. 3B, left graph; Student’s *t* test, *p* = 0.006; Table 1, f; Fig. 3C), but there are no genotype differences in TH-positive cell counts in the SNc (Fig. 3B, right graph; Table 1, f). These data show that there is an early, enduring increase in the number of TH-positive neurons in *Cdon^−/−^* mice compared with WT littermates. Interestingly, this increase is selective to the medial portion (i.e., VTA region) of the midbrain dopamine somatodendritic region.

### Selective increase in dopamine levels in the PFC of adult *Cdon^−/−^* mice

**Figure 4.**
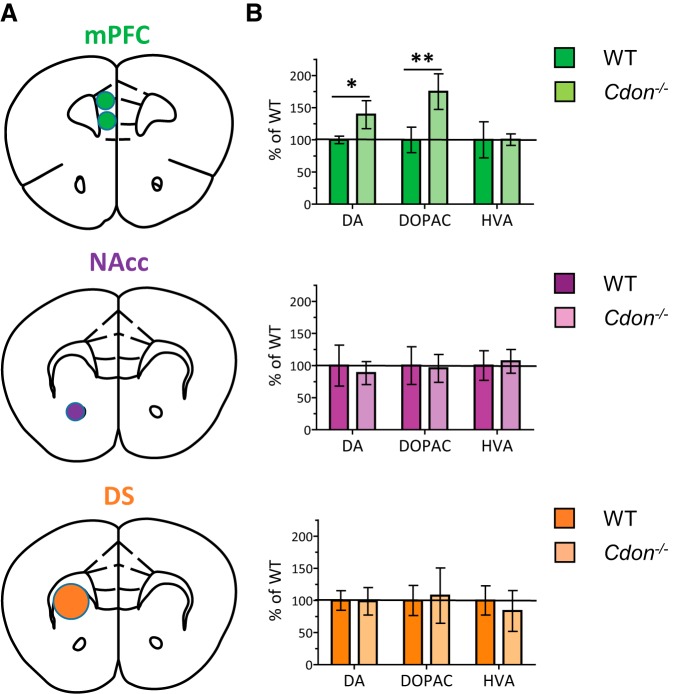
Greater dopamine and DOPAC concentrations in the mPFC, but not the NAcc or DS, of adult *Cdon^−/−^* mice. ***A***, Brain samples were taken from each target region illustrated. ***B***, HPLC revealed a selective increase in the dopamine and DOPAC concentrations of the mPFC of *Cdon^−/−^* mice, an effect that was not seen in the NAcc or DS (Table 1, g). *n* = 7-10 animals/group. *p < 0.05, **p < 0.01.

To examine whether the increase in the number of TH-positive neurons in the VTA is associated with differential content of dopamine and the dopamine metabolites DOPAC and HVA in forebrain terminal regions, we conducted HPLC on tissue samples of VTA and SNc targets: mPFC, NAcc, and DS (Fig. 4A, illustrations). As shown in Figure 4B (top), in the mPFC the levels of dopamine and DOPAC in *Cdon^−/−^* mice are significantly elevated compared to WT littermates (Student’s *t* test with Holm–Bonferroni correction: dopamine, *p* = 0.03; DOPAC, *p* = 0.019; Table 1, g). In contrast, there were no differences between genotypes in the concentrations of dopamine, DOPAC, and HVA in the NAcc (Fig. 4B, middle; Table 1, g) or DS (Fig. 4B, bottom; Table 1, g). These findings suggest that the increase in the number of TH-positive cells in the VTA of *Cdon^−/−^* mice is specific to VTA dopamine neurons that project to the mPFC.

### Increased number of TH-positive varicosities in the mPFC of *Cdon^−/−^* mice

**Figure 5.**
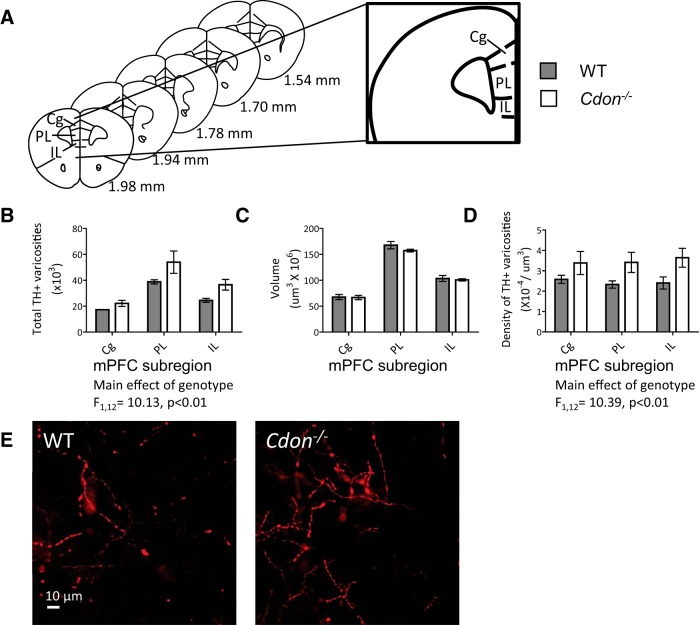
Increased number of dopamine varicosities in the mPFC of *Cdon^−/−^* mice. ***A***, Stereological quantifications of the number of dopamine varicosities in the Cg, the PL, and the IL pregenual mPFC. ***B***, The total number of dopamine varicosities was greater in the *Cdon^−/−^* mice compared with WT controls (ANOVA_Genotype_, *p* = 0.0079; Table 1, h). ***C***, There were no differences in the volume that dopamine varicosities occupied in the mPFC between *Cdon^−/−^* and WT mice (Table 1, i). ***D***, Likewise, an increase in the density of dopamine varicosities was observed in all three subregions (ANOVA_Genotype_, *p* = 0.0073; Table 1, j). ***E***, Representative photomicrographs at high magnification illustrating differences in the total number/density of dopamine varicosities in the PL mPFC comparing *Cdon^−/−^* and WT mice. *n* = 3 mice/group.

We then performed stereological quantifications of dopamine varicosities in the Cg, PL, and IL subregions (Fig. 5A) of the pregenual mPFC. We found a significant increase in the total number of dopamine varicosities (i.e. dopamine presynaptic sites) in the Cg, PL, and IL subregions of the mPFC of *Cdon^−/−^* mice compared with controls (Fig. 5B; ANOVA_Genotype_, *p* = 0.0079; Table 1, h). To determine whether this increase in the total number of dopamine presynaptic sites results from an enhanced expanse of the dopamine innervation to the mPFC, we quantified the volume of the dopamine input to each subregion using the Cavalieri method (Manitt et al., 2011; Reynolds et al., 2015). There were no differences in dopamine input volume between genotypes in any of the subregions examined, indicating that dopamine axons in *Cdon^−/−^* mice are not extending to other mPFC layers (Fig. 5C, ANOVA_Genotype_; Table 1, i). This led to a significant increase in the density of dopamine varicosities in all three subregions (Fig. 5D; ANOVA_Genotype_, *p* = 0.0073; Table 1, j), which could be seen at high magnification (Fig. 5E).

### Locomotor activity of *Cdon^−/−^* mice reveals an attenuation of behavioral plasticity in adulthood

**Figure 6.**
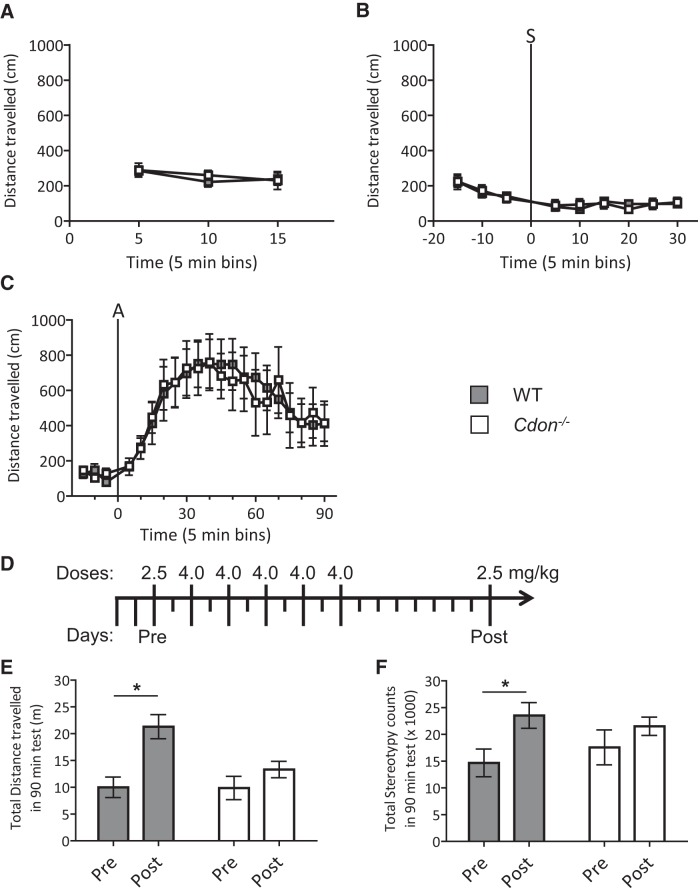
Locomotor activity testing of *Cdon^−/−^* mice reveals attenuation of behavioral plasticity in adulthood. ***A–C***, First exposure/habituation to the locomotor testing environment (***A***), habituation to handling and saline injection (injection denoted by “S” vertical line; ***B***), and the first injection of amphetamine (injection denoted by “A” vertical line, 2.5 mg/kg, i.p.; ***C***) all produce indistinguishable levels of locomotor activity between *Cdon^−/−^* and WT controls (Table 1, k, l, and m, respectively). ***D***, ***E***, In contrast, a sensitizing schedule of amphetamine injections (***D***) induced robust locomotor sensitization in WT controls (***E***), while locomotor sensitization in *Cdon^−/−^* mice was greatly attenuated (ANOVA_Genotype×Time_, *p* = 0.043; Table 1, n). ***F***, Stereotypy counts were increased in WT controls, but did not change significantly in *Cdon^−/−^* mice. *n* = 6-10 animals/group.

To examine the possible consequences of the neuroanatomical changes that we observed in the VTA and mPFC of *Cdon^−/−^* mice, we evaluated the locomotor responses of adult *Cdon^−/−^* and WT mice. Both genotypes exhibited similar levels of locomotor activity when placed in the novel locomotor testing environment (Fig. 6A; Table 1, k) and in response to a saline injection (Fig. 6B; Table 1, l). *Cdon^−/−^* and WT mice also responded identically to the first dose of d-amphetamine (2.5 mg/kg; Fig. 6C; Table 1, m). Thus, *Cdon^−/−^* and WT littermates respond with similar amounts of locomotor activity in response to novelty and to single exposure to a stressor (e.g., saline injection) or a stimulant drug of abuse (e.g., amphetamine).

The amount of locomotor activity typically increases with repeated drug experience, a phenomenon known as sensitization. In order to test locomotor sensitization, mice were given 5 doses of 4 mg/kg d-amphetamine every other day (over the next 2.5 weeks) and then were left undisturbed in their home cage for 8 d (Fig. 6D, schedule). Mice were then tested at the same 2.5 mg/kg dose that was used in the first trial, ∼3 weeks previously. WT mice exhibited robust locomotor sensitization, and when pre- and postsensitization levels were compared, the amount of locomotor activity nearly doubled (Fig. 6E; within-subjects Bonferroni’s *post hoc* test on WT, *p* = 0.0018; Table 1, n). In contrast, no change in the amount of locomotor activity was observed in the *Cdon^−/−^* mice when pre- and postsensitization levels were compared (Fig. 6E). Of note, drug-induced stereotypy (repetitive behavior) was also increased in WT mice over time (Fig. 6F, within-subjects Bonferroni’s *post hoc* test on WT, *p* = 0.027; Table 1, o), but did not change significantly in *Cdon^−/−^* mice (Fig. 6F). These data demonstrate that while baseline locomotor responses to stress and to an initial dose of amphetamine were indistinguishable between *Cdon^−/−^* and WT mice, amphetamine-induced behavioral plasticity is attenuated in *Cdon^−/−^* mice.

### Attenuated sensorimotor gating function in adult *Cdon^−/−^* mice

**Figure 7.**
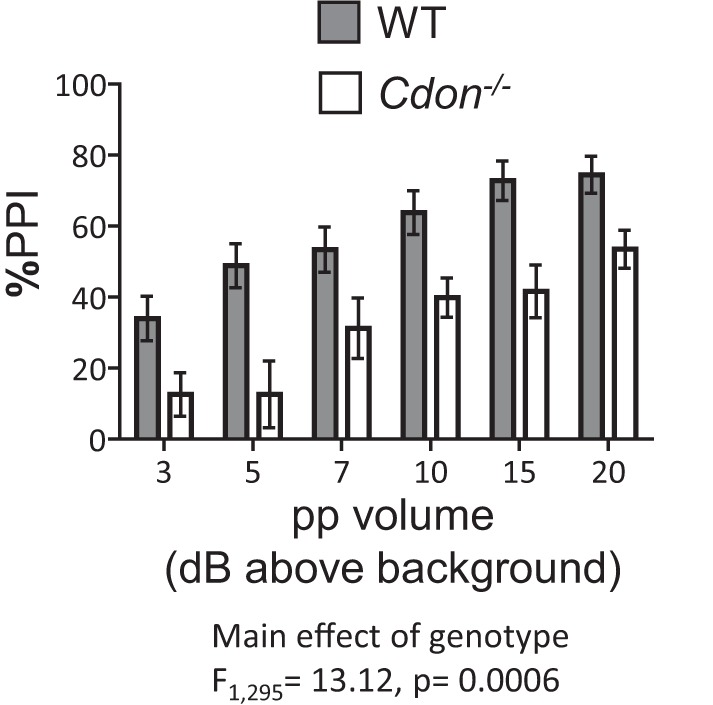
Sensorimotor gating function is attenuated in adult *Cdon^−/−^* mice. PPI is measured relative to the baseline startle for each mouse and is shown according to the volume of each prepulse (pp3, pp5, pp7, pp10, pp15, pp20), which is the number of decibels above environmental white noise (70 dB). The PPI percentage was calculated for each prepulse volume (mean prepulse) as a percentage of the unsignaled startle intensity (mean startle) for each individual mouse, and the baseline movement in the absence of acoustic pulses (mean null) was subtracted from all values: PPI% = 1 − (mean prepulse − mean null)/(mean startle − mean null)*100. When the normalized PPI for each individual were compared by two-way ANOVA_ppvolume × Genotype_ significant effects of volume (ANOVA_ppvolume_, *p* < 0.0001; Table 1, p) and genotype (ANOVA_Genotype_, *p* < 0.001; Table 1, p) were observed on PPI.

To further examine behavioral consequences of the neuroanatomical changes that we observed in *Cdon^−/−^* mice, we next tested sensorimotor gating function in adult mice, which can be modulated by alterations in mesocortical dopamine function (Swerdlow et al., 1990; Tenn et al., 2005; Grant et al., 2007). Rodents startle in response to loud noises, and this reflex is typically reduced if an acoustic prepulse is given. The reduction in the startle magnitude is called PPI, and louder prepulses typically produce greater PPI. As expected in WT mice, increasing the prepulse volume increases PPI (Fig. 7; main effect of ppvolume, *p* = 0.0001; Table 1, p). However, PPI was significantly reduced in *Cdon^−/−^* mice compared with WT littermates (Fig. 7; main effect of genotype, *p* = 0.0006; Table 1, p).

## Discussion

In this study, we assessed the role of the Shh receptor Cdon in the development of VTA and SNc dopamine neurons. We found that Cdon is expressed in the proliferating progenitor zone of the embryonic ventral midbrain, and that the number of proliferating cells in this region is increased in *Cdon^−/−^* embryos. These findings indicate that Cdon is involved in the regulation of neuronal proliferation in progenitors of the ventral midbrain. Consistent with this idea, we found that the number of TH-positive neurons is increased in the VTA of *Cdon^−/−^* mice at birth and that this effect endures into adulthood. In contrast, the number of TH-positive neurons in the SNc is not significantly altered in *Cdon^−/−^* mice at either age. In accordance with an increase in the number of mesocortical VTA dopaminergic neurons, there is a greater number of dopamine presynaptic sites in the mPFC, and corresponding increases in baseline concentrations of dopamine and dopamine metabolites selectively in this region in adult *Cdon^−/−^* mice. These data indicate that Cdon is selectively involved in the development of mesocortical dopamine neurons. Finally, we found that adult *Cdon^−/−^* mice fail to exhibit dopamine-dependent behavioral plasticity in response to repeated injections of amphetamine. Based on these data, we suggest that Cdon plays an important role in the encoding of diversity within the population of dopamine neurons of the midbrain, influencing both the development of the mesocortical dopamine pathway as well as behavioral outputs that involve this neural circuitry.

### Cdon and dopaminergic neuron development

In the first phase of dopamine neuron specification, notochord-derived Shh initiates the specification of the ventral midbrain. Inactivation of Shh signaling at this phase leads to almost complete absence of dopaminergic neurons (Blaess et al., 2006). During the second phase, Shh is expressed by dopaminergic neuron progenitors and the duration of its expression contributes to their fate decisions into dopamine neurons and their segregation between VTA and SNc (Blaess et al., 2011; Hayes et al., 2011). Accordingly, inactivation of Shh signaling only after Shh is expressed within the dopamine progenitors (i.e., inactivation only during the second phase) leads to a VTA-specific increase in the number of dopamine cells, leaving the number of SNc dopamine cells unchanged (Hayes et al., 2013). Interestingly, this phenotype is very similar to what we observed in *Cdon^−/−^* mice, where we observed an increase in VTA dopaminergic neurons, but no change in SNc neurons. These results support the idea that the main role of Cdon is in the second phase of dopaminergic neuron induction. In agreement with this, we did not observe a difference in the number of TH-expressing neurons at E12.5, further indicating that Cdon plays a minor role, if any, in the first phase of dopaminergic neuron induction.

A previous study tested the importance of continued Shh expression in dopamine neurons in adult mice. Gonzalez-Reyes et al. (2012) used a Cre-Lox recombination strategy in order to selectively remove Shh from neurons that express the dopamine transporter. The dopamine transporter is a marker of mature dopamine neurons, and when Shh was removed from these neurons, premature degeneration was observed in the dopamine neurons of the SNc (Gonzalez-Reyes et al., 2012). Therefore, continued Shh expression is critical for the long-term maintenance of dopamine neurons in the SNc and nigrostriatal circuitry. Because we do not observe similar degeneration in the *Cdon^−/−^* mice, we propose that this Shh effect on adult SNc circuitry may not require Cdon.

A previous report described a reduction in the number of TH-positive cells in E13.5 *Cdon^−/−^* embryos (Kwon et al., 2014). One possible reason for this discrepancy with our data is the presence or absence of HPE in the *Cdon^−/−^* embryos analyzed. HPE is a condition that results in inadequate formation of the neural midline and ventricle malformation. Many studies have reported *Cdon^−/−^* mouse lines with as many as ∼80% of mutants showing HPE at birth, with virtually none surviving into adulthood (Cole and Krauss, 2003; Zhang et al., 2006, 2011; Bae et al., 2011; Hong and Krauss, 2013). In contrast, our *Cdon* mouse line exhibits a lower rate of HPE (∼10-20%), and only mice that did not show any obvious signs of HPE and that remained healthy into adulthood were included in our study. This variability between studies is in part attributed to the fact that the expression of HPE in *Cdon^−/−^* mice depends strongly on the genetic background and genetic modifiers of this receptor (Cole and Krauss, 2003; Zhang et al., 2006; Bae et al., 2011). Importantly, HPE has indeed been associated with decreased proliferation in primary neuronal cultures in *Cdon* mutant mice (Zhang et al., 2006). Therefore, when present, HPE could potentially be acting in opposition to the enhanced proliferation phenotype that we observed in our study.

The increased numbers of Ki67-positive cells that we observe at E12.5 coincides with the second stage of Shh signaling. At this stage in development, it would appear that Cdon modulates the proliferation rate of dopamine neuron progenitors. Indeed, it has been shown that once dopamine neuron progenitors begin to express Shh, the duration and timing of Shh expression contributes to fate decisions made by these cells (Blaess et al., 2011; Hayes et al., 2011). Therefore, mechanisms that alter the intensity or the duration of Shh signaling and expression are likely modified by removing the Shh receptor Cdon. This could result in increased numbers of proliferating dopamine neurons that go on to contribute mainly to the mesocortical pool.

An increasing number of reports show that dopamine neurons in the VTA are a heterogeneous population, and that the neuroanatomical, electrophysiological, and developmental properties of these neurons are dictated by the targets they innervate. It is therefore possible that in the midbrain, only a subset of the medial portion of the ventral midbrain dopamine neuron progenitors coexpress Cdon and Shh, namely those dopamine progenitors that are fated to innervate the mPFC. Increased numbers of mesocortical dopamine neurons would presumably lead to increased dopamine input to and dopamine concentrations in the mPFC. Because the dopamine innervation to the mPFC is a protracted event, which extends into early adulthood, we would also predict that these effects will only manifest fully in adulthood. The fact that *Cdon^−/−^* mice exhibit a greater number of presynaptic dopamine sites in the mPFC without showing increases in the expanse that dopamine fibers occupy in this regions indicates that Cdon plays a role in the proliferation of mesocortical dopamine neurons, but not in their guidance toward forebrain targets.

### Cdon, mesocortical dopamine, and behavioral responses to drugs of abuse

Locomotor responses to amphetamine depend mainly on drug-induced dopamine release in the NAcc (Vezina et al., 1991; Vezina, 1993), which is influenced by dopamine function in the mPFC (Bimpisidis et al., 2013). For example, mice that are haploinsufficient for the Netrin-1 receptor *Dcc* exhibit increased baseline concentrations of dopamine and dopamine metabolites in mPFC, which in turn causes blunted amphetamine-induced dopamine release in the NAcc (Flores et al., 2005; Pokinko et al., 2015). Interestingly, adult *Dcc* haploinsufficient mice also fail to show sensitization to the locomotor effects of amphetamine upon repeated exposure (Flores et al., 2005; Grant et al., 2007; Yetnikoff et al., 2010). Thus, it is likely that the behavioral changes observed in adult *Cdon^−/−^* mice could result from blunted responsiveness of NAcc-projecting dopamine neurons, which are associated with increased mPFC dopamine function (Bimpisidis et al., 2013).

The selective effects of Cdon on the mesocortical dopamine projections are particularly interesting in light of another recent study that also highlighted the sensitivity of this circuit to changes in Shh signaling (Kabanova et al., 2015). Gli2 is a transcription factor mediating many of the intracellular effects of Shh signaling in the brain (Vokes et al., 2007), and it is involved in the specification of dopamine neurons (Matise et al., 1998). Recently, Gli2 was conditionally removed from the cells of the ventral midbrain through En1-Cre-induced recombination (Kabanova et al., 2015). In these mice, dopamine levels were decreased in the mPFC, but not in the NAcc (Kabanova et al., 2015). Tracing experiments also demonstrated that the density of dopamine projection into the mPFC was reduced, whereas the density of dopamine fibers in the NAcc was not altered (Kabanova et al., 2015). While novel object learning was unimpaired in these mice, Kabanova et al. (2015) report increases in the amount of perseverative behavior during a five-choice serial reaction time task. This deficit in attention processing may be linked to the alterations in mPFC dopamine circuitry that we observe in adult *Cdon^−/−^* mice (Moghaddam, 2002; Grace et al., 2007).

The relationship between increased dopamine concentrations in the mPFC and impaired PPI in *Cdon^−/−^* mice is surprising, and at this point we cannot provide a conclusive mechanistic explanation of this finding. Deficits in baseline prepulse inhibition have been shown to result from reduced mesocortical dopamine function (Bubser and Koch, 1994; Swerdlow and Geyer, 1998; Kohl et al., 2013). Furthermore, because mesocortical dopamine function and responsiveness of mesolimbic dopamine neurons to stressors and drugs of abuse are inversely related (Jackson and Moghaddam, 2001; Ventura et al., 2004; Scornaiencki et al., 2009; Pokinko et al., 2015), it has been suggested that the role of mPFC dopamine on PPI is mediated by changes in ventral striatal dopamine function (Bubser and Koch, 1994; Koch and Bubser, 1994; Ellenbroek et al., 1996; Grant et al., 2007; Flores, 2011). However, there are no differences in nucleus accumbens dopamine concentrations between *Cdon^−/−^* and WT mice. It is possible that impaired sensorimotor gating function in *Cdon^−/−^* mice results from alterations in mPFC and/or nucleus accumbens dopamine release that could only be captured via *in vivo* microdialysis or voltammetry. Moreover, it is also possible that either insufficient or excessive extracellular dopamine concentration in the mPFC lead to deficits in PPI as has been shown for the effects of mPFC dopamine function on cognitive processing (Floresco, 2013). Future studies will be aimed at addressing this issue by directly using neurochemical and lesion approaches used in previous studies (Jackson and Moghaddam, 2001; Ventura et al., 2004; Grant et al., 2007; Scornaiencki et al., 2009; Pokinko et al., 2015).

In conclusion, it is increasingly becoming clear that the diversity of midbrain dopamine neurons results from developmental processes that determine the heterogeneity of these cells, potentially long before this diversity can be accurately described (Anderegg et al., 2015). This diversity can be captured by comparing anatomical and functional properties of VTA and SNc dopamine neurons, but also by comparing electrophysiological properties of dopamine projections to cortical versus limbic targets (Lammel et al., 2008; Roeper, 2013), both of which are impossible to capture at early embryonic stages. In the current study, we demonstrate that the Shh receptor Cdon plays a specific role in the developmental organization and function of the mesocortical dopamine pathway. These changes also influence adult behavioral responses to drugs of abuse and sensorimotor gating. Our data therefore provide novel insights toward the diverse consequences of alterations in Shh signaling and describe changes in the VTA that have potential implications for psychopathologies such as schizophrenia (Meyer et al., 2008; Boyd et al., 2015) and attention deficit hyperactivity disorder (Heussler et al., 2002).

## Synthesis

The decision was a result of the Reviewing Editor Karen Szumlinski and the peer reviewers coming together and discussing their recommendations until a consensus was reached. A fact-based synthesis statement explaining their decision and outlining what is needed to prepare a revision is listed below. The following reviewers agreed to reveal their identity: Stephen Rayport, Stefano Puglisi-Allegra

Upon review of the revised article, the 2 expert reviewers and I agree that the report is much improved with respect to the main text. However, there still remains a number of issues with the display items that will require editorial re-review before a final decision can be rendered on the article. Both reviewers have highlighted these dislay item issues.

Reviewer #1:

The authors have responded cogently to the initial reviews.

However, the figures require significant attention.

Figure 1: Panel letters (A, B, C...) and panel labels (DAPI/Ki67/TH) are all in 18 pt; panel letters should be the largest, then panel labels, e.g. 15 pt.. Scale bar lacks calibration; better to put the "100 um" on the figure itself. Caption would be improved with some reporting of what is being shown.

Figure 2: Panel letters and labels are all in 18 pt. Panel F: It is misleading to provide bars that are NS; better to remove.

Figure 3: Panel letters, labels, and anatomical division labels (VTA, SN) are all in 18 pt, and should be hierarchical. Panel C, the panel labels need to be smaller. In the caption, the use of "significant increases" when describing observations in adulthood implies that there is continued proliferation of dopamine neurons; it would be better to say that there are greater numbers of dopamine neurons.

Figure 4: Similarly, "selective increase" in adult mice suggests continued increase, when what was measured was the value in adulthood, so it would be better to use "greater" or "larger" or "is greater in adulthood."

Figure 5: Panels B and D: the statistics should be in the captions (the asterisks do not refer to anything). Panel E: The monochromatic micrographs are hard to see; they should be brightened up, or converted to white on black to improve visibility.

Figure 6: Panels B and C: the injections (vertical lines) needs labels, e.g. vehicle and amphetamine. Panel D: fonts are overall too large. Panel E and F: since previous usage has been to provide a horizontal bar indicating the comparison showing statistical significance, the same should be added here (i.e. a bar over pre vs post in the WT mice).

Figure 7: The asterisks are misused since the statistic is an overall genotypic effect (ANOVA) and individual t-tests are not reported.

Reviewer #2:

All comments have been adequately addressed.

Although I have not been able to find the table 1 in R1 text, I trust that the table reports the items described in the text.
